# RBP2 Promotes Adult Acute Lymphoblastic Leukemia by Upregulating BCL2

**DOI:** 10.1371/journal.pone.0152142

**Published:** 2016-03-23

**Authors:** Xiaoming Wang, Minran Zhou, Yue Fu, Ting Sun, Jin Chen, Xuemei Qin, Yuan Yu, Jihui Jia, Chunyan Chen

**Affiliations:** 1 Department of Hematology, Qilu Hospital, Shandong University, Jinan, Shandong, P. R. China; 2 Anhui Medical College, Hefei, Anhui, P. R. China; 3 Department of Microbiology/Key Laboratory for Experimental Teratology of Chinese Ministry of Education, School of Medicine, Shandong University, Jinan, Shandong, P. R. China; Pennsylvania State University, UNITED STATES

## Abstract

Despite recent increases in the cure rate of acute lymphoblastic leukemia (ALL), adult ALL remains a high-risk disease that exhibits a high relapse rate. In this study, we found that the histone demethylase retinoblastoma binding protein-2 (RBP2) was overexpressed in both on-going and relapse cases of adult ALL, which revealed that RBP2 overexpression was not only involved in the pathogenesis of ALL but that its overexpression might also be related to relapse of the disease. RBP2 knockdown induced apoptosis and attenuated leukemic cell viability. Our results demonstrated that BCL2 is a novel target of RBP2 and supported the notion of RBP2 being a regulator of BCL2 expression via directly binding to its promoter. As the role of RBP2 in regulating apoptosis was confirmed, RBP2 overexpression and activation of BCL2 might play important roles in ALL development and progression.

## Introduction

Acute lymphoblastic leukemia (ALL), a clonal malignancy of lymphocyte precursors, is a life-threatening neoplasm characterized by uncontrolled growth and leukemic expansion of immature lymphoblastic progenitor cells [1–3]. This disease is prevalent in both children and adults, with a peak incidence between the ages of 2 to 5 years, and constitutes 15% of adult leukemias [1, 4]. Multi-agent treatment regimens can cure more than 80% of children diagnosed with ALL. In contrast, the prognosis for adults with ALL is significantly worse and worsens with increasing age [1, 4, 5].

ALL is a heterogeneous disease with multiple, prognostically relevant genetic aberrations [1]. For many years, leukemia was thought to stem primarily from genetic alterations, which affect proliferation, differentiation, apoptosis and gene transcription of leukemic cells. In recent years, epigenetic alterations have also been recognized as a major driving force in the development of ALL [6, 7]. Aberrant epigenetic lesions, particularly DNA methylation and miRNA dysregulation are common in ALL. MicroRNA-193b-3p acts as a tumor suppressor in T-cell acute lymphoblastic leukemia [8], and miR-17~92 is downregulated in BCR-ABL-positive human ALL samples [9]. 5-aza-2′-deoxycytidine (decitabine) has been used to target hypermethylation clinically [10, 11]. Therefore, the study of histone demethylase as another epigenetic modification in leukemia urgently needs to be explored. Recently, the histone 3 lysine 27 (H3K27) demethylases JMJD3 and UTX have been found to play contrasting roles in ALL [12, 13].

RBP2 is a newly identified member of the JARID protein family, which accounts for histone demethylase (HDM) activity. RBP2 specifically targets tri- and di-methylated lysine 4 of histone H3 (H3-K4) for demethylation [14]. RBP2 is also referred to as KDM5A or JARID1A. This protein was originally identified as a critical retinoblastoma protein (pRB)-binding protein [14, 15]. Recently, RBP2 was identified as one chromatin-modifying enzyme that participates in the carcinogenesis and progression of human cancers such as lung cancer [16], gastric cancer [17–20] and breast cancer [21]. We have shown that RBP2 promoted the initiation and progression of gastric cancer. Whether RBP2 plays a key role in ALL remains to be determined.

B-cell lymphoma 2 (BCL2), a well-established inhibitor of mitochondrial apoptotic pathways, has emerged as a potential therapeutic target in both leukemia and solid tumors [22, 23]. In BCR-ABL-positive ALL, BCL2 knockdown resulted in strong induction of apoptosis, and BCL2 is identified as a potential therapeutic target [9]. To date, inhibitors targeting BCL2 have been explored in clinical trials. Here, we aim to determine the role of RBP2 in ALL. In this study, we showed that RBP2 promoted ALL by directly upregulating BCL2.

## Materials and Methods

### Ethics statement

This study was approved by the Ethics Committee of the School of Medicine of Shandong University. The patients or their next of kin provided written informed consent for participation in this study.

### Patient samples

Bone marrow samples were obtained from patients at the Department of Hematology, Qilu Hospital of Shandong University, Jinan, China,who had newly diagnosed ALL (*de novo* ALL, n = 42), ALL with complete remission (ALL-CR, n = 36), relapsed ALL (ALL-relapse, n = 9) and iron deficiency anemia (n = 33). All of the patients were treated at Qilu Hospital between January, 2012 and June, 2015. Patients were included based on the following inclusion criteria: The 42 cases of *de novo* ALL patients were newly diagnosed with common ALL based on the World Health Organization(WHO) Classification of Tumors- Pathology and Genetics of Tumors of Hematopoietic and Lymphoid Tissue (2008)and were included prior to treatment. The 36 cases of ALL-CR patients all received complete remission after standard chemotherapy, and their bone marrow examinations showed ≤5% primary cells. The relapse was confirmed by bone marrow examination if the clinical symptoms reappeared after complete remission. Patients with iron deficiency anemia and without abnormal hematopoiesis of the bone marrow were included as controls. The clinical features of all of the patients are summarized in S1 Table.

### Sample preparation

Mononuclear cells were isolated from samples by Ficoll-Hypaque density-gradient centrifugation then cryo-preserved in a −80°C freezer for subsequent experiments.

### Immunofluorescence (IF)

Mononuclear cells isolated from patient bone marrow samples were used to prepare cytospins with glass slides treated by Poly-L-lysine (PLL) and then fixed in cold acetone. After fixation, mononuclear cells were briefly washed with 0.5% Triton X-100 in phosphate buffered saline (PBS) for 10 min; non-specific binding was then blocked by incubation for 1 h in 5% goat serumsealing fluid. The samples were incubated with the primary antibody (anti-RBP2 antibody,1:100, Abcam) overnight at 4°C. The mononuclear cells were then washed three times for 5 min in PBS, followed by incubation with their respective Alexa Fluor-conjugated secondary antibodies (goat anti-rabbit IgG, 1:1000,CST) for 1 h in the dark. After four washes of 5 min each, mononuclear cells were stained with 4', 6-diamidino-2-phenylindole (DAPI) for 5 min. Slides were examined by confocal laser scanning microscopy.

### RNA extraction and quantitative real-time PCR (qRT-PCR)

Total RNA from human bone marrow samples and Jurkat cells was extracted using Trizol reagent (Invitrogen, Carlsbad, CA, USA). The extracted RNA was then reverse-transcribed using a RevertAid First Strand DNA Synthesis (RT) kit (Fermentas Life Science, Canada). Gene expression was verified by PCR using the TaqMan gene expression assay kit (Life Technologies, USA) or the SYBR Premix Ex Taq kit (Takara, Japan) with β-actin as a control. The probes for RBP2 (Applied Biosystems) were Hs00231908_m1. The mRNA level of BCL2 was determined by RT and SYBR-Green real-time PCR assays (TaKaRa, Japan). Gene expression was normalized to that of β-actin. Expression was calculated using the 2^-ΔΔCT^method. The sequences of the primers used are as follows: β-actin, 5’-AGTTGCGTTACACCCTTTCTTG-3’ and 5’-CACCTTCACCGTTCCAGTTTT-3’; BCL2, 5’-GGTGAACTGGGGGAGGATTG-3’and 5’-GTGCCGGTTCAGGTACTCAG-3’.

### Chromatin immunoprecipitation (ChIP)

For the ChIP assay, a Millipore ChIP assay Kit was used to treat the prepared Jurkat cells according to the kit protocol. The Jurkat cells were cross-linked by incubation in 37% formaldehyde solution for 10 min at 37°C and sonicated to develop soluble chromatin with DNA fragments ranging in size from 200 to 800 bp. DNA was purified from the chromatin fragments immunoprecipitated with antibodies against RBP2 (Abcam) and used for PCR amplification. The PCR primers for the BCL2 promoter were: BCL2-promoter-a, 5’ CCCACATACACGGCTAGAAAAGG-3’ and 5’-CCATGAAAACAAGGGCTGGAAAA-3; BCL2-promoter-b, 5’-ACCCCAGCGACCACCAA-3’ and 5’-AAAGAGCCCTCCTCTGAGCC-3’; BCL2-promoter-c, 5’-AGGAGGGCTCTTTCTTTCTTCTTT-3’ and 5’-CGGCACCTTCGCTGGCA-3’.

### Western blotting

Cells were collected by centrifugation, washed twice in PBS and lysed for 30 min on ice in DTT-buffer supplemented with 1 mM PMSF. Total cellular proteins were separated by SDS-PAGE and transferred to PVDF membranes, which were probed with antibodies against RBP2 (1:400, Abcam), BCL2 (1:250, Santa Cruz Biotechnology) and β-actin (1:10000, Sigma) overnight at 4°C followed by horseradish peroxidase-labeled goat-anti-rabbit IgG (1:6000, Abcam) for 50 min. Antigens were revealed using Enhanced Chemiluminescence Reaction (ECL+, Millipore, USA). For western blot analysis of histone H3K4 di- and tri-methylation, a total histone fraction was isolated from nuclei by dilute acid extraction. The membranes were incubated with the antibodies against di- and tri-methylated H3K4 (1:3000, Abcam). H3 was used as a loading control (1:10000, Abcam).

### Cell culture and siRNA interference

Jurkat cells were maintained in our laboratory. All cell lines were grown in RPMI1640 medium (Gibco, USA) supplemented with 10% fetal bovine serum (Gibco, USA) without antibiotics. Cell lines were incubated in a humidified atmosphere containing 5% CO2 at 37°C. Cells were transfected with RBP2 siRNA (Invitrogen, Carlsbad, CA, USA) using Lipofectamine 2000 (Invitrogen, Carlsbad, CA, USA). The sequences for RBP2 and its control siRNA were 5'- CCA GCA CCA CCU CCU UCC UUC AUA A -3' and 5'- CCU ACA UCC CGA UCG AUG AUG UUG A -3', respectively.

### Cell proliferation

We used 5-ethynyl-2’-deoxyuridine (EdU) assay to detect the proliferative rate of Jurkat cells. Transfected cells were incubated with EdU for 2 h before fluorescent detection. Treated cells were used to prepare cytospins with glass slides, fixed with 4% paraformaldehyde for 30 minutes, and then stained using the Cell-Light™ EdU Apollo®488 In Vitro Imaging Kit (RioBio, China) according to the manufacturer’s instructions. Slides were examined by confocal laser scanning microscopy.

### Soft agar assay

In brief, 1 ml of 1% agar in complete 2☓DMEM containing 20% fetal bovine serum (FBS) was plated as the basal layer in 6-well plates. Cells in complete medium containing 0.4% agar were seeded on the basal layer. Plates were incubated at 37°C in a CO2 incubator for 21 days. Dense colonies were microscopically examined and counted on the 21st day.

### Flow cytometry

Control and RBP2-depleted cells were used for labeling of apoptotic cells. Cells were harvested after transfection, centrifuged and resuspended in binding buffer. Then, cells (100 μL of the cell suspension solution) were stained by 7-AAD-PE, incubated at room temperature for 15 min, and then subjected to flow cytometry (Becton Dickinson).

### Luciferase reporter assay

The BCL2 reporter construct harboring its promoter sequences was gifted by Addgene (#15381).We transfected Jurkat cells with RBP2-siRNA on day 1 and with the wild-type BCL2 promoter reporter plasmid on the following day. The thymidine kinase promoter was cotransfected to monitor transfection efficiency. After 48 h, luciferase activity was determined using the Dual-Luciferase Reporter Assay System (Promega). Luciferase activity of the BCL2 promoter reporter was normalized to thymidine kinase renilla activity.

### Statistical analysis

The data obtained from biological replicates were presented as the means (±SD or SEM). Student’s t-test, Mann-Whitney U-test, one-way ANOVA and Pearson correlation efficiency were used to analyze the differences between different groups using GraphPad Prism for Windows version 5.00 (GraphPad Software, La Jolla, CA, USA). P < 0.05 was considered statistically significant.

## Results

### RBP2 is highly expressed in adult ALL

**Fig 1:**
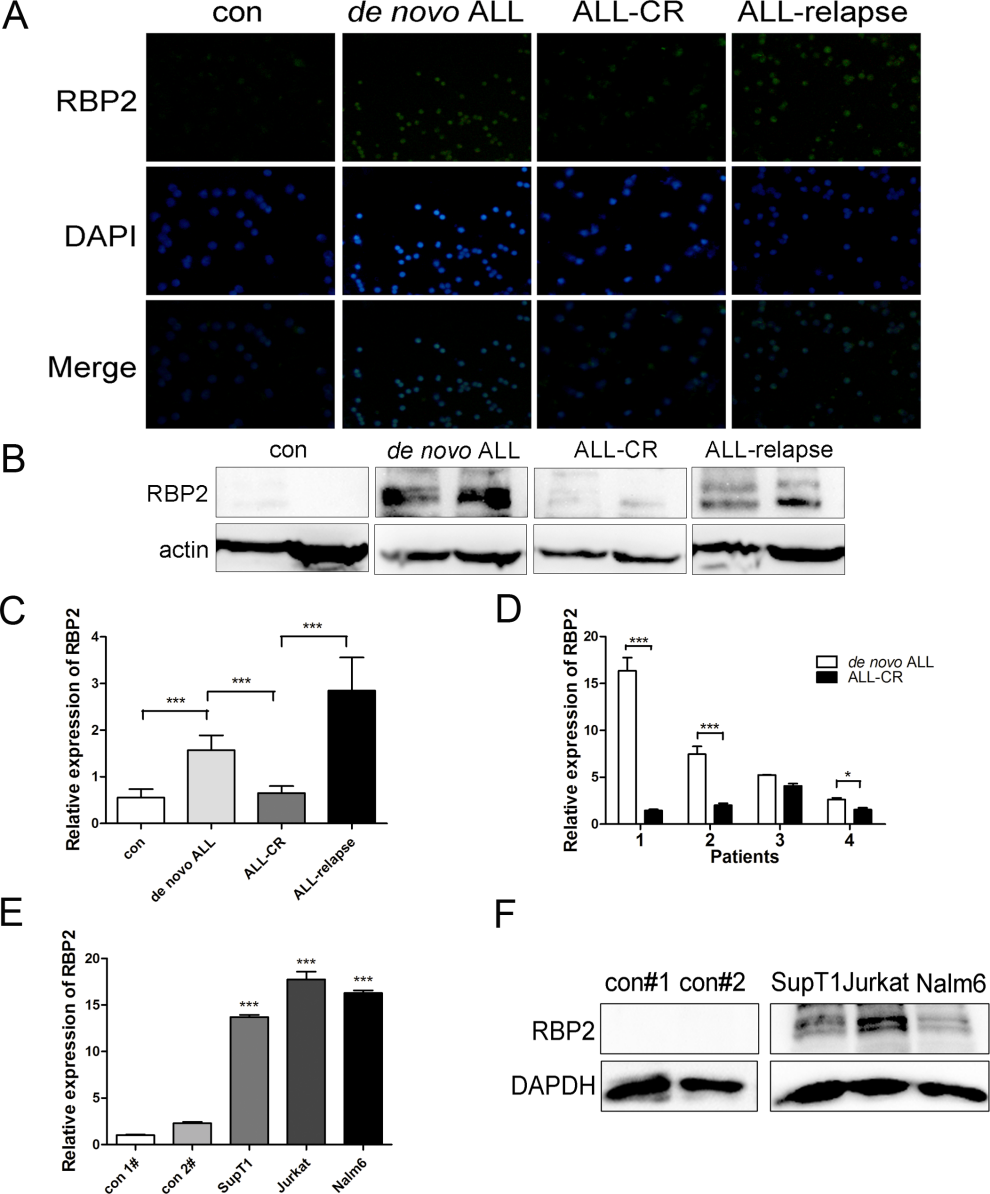
RBP2 is highly expressed in ALL samples and ALL cell lines. (A, B) Immunofluorescence and western blotting analysis of RBP2 protein levels in bone marrow samples obtained from ALL patients. (C) RBP2 mRNA levels of bone marrow samples from 42 *de novo* ALL, 36 ALL-CR and 9 ALL-relapse patients were analyzed by qRT-PCR. Mann-Whitney U-test was used to calculate the P value. (D) Relative expression of RBP2 mRNA in 4 matched ALL patients. *P< 0.05, ***P< 0.001 by Student’s t-test. (E) qRT-PCR analysis of relative expression of RBP2 mRNA in ALL cell lines and bone marrow samples from healthy patients. ***P< 0.001 by Student’s t-test: ALL cell lines vs. controls. (F) Western blotting analysis of RBP2 protein levels in ALL cell lines and bone marrow samples from healthy patients. The results were confirmed in 3 independent experiments.

To determine the potential role of RBP2 in ALL, we investigated the expression of RBP2 in bone marrow samples from 42 newly diagnosed adult patients (*de novo* ALL), 36 patients in complete remission (ALL-CR) and 9 relapsed adult patients (ALL-relapse). The clinical characteristics of ALL patients are listed in S1 Table. After measuring RBP2 protein levels with immunofluorescence and western blotting, we found that RBP2 was uniformly expressed at high levels in *de nov*o ALL and relapsed patients. Upon complete remission, RBP2 was reduced to normal levels (Fig 1A and 1B). RBP2 mRNA expression levels were consistent with the protein results. *De novo* ALL samples revealed approximately 2-fold higher RBP2 mRNA levels than controls, and ALL-relapsed patients had the highest RBP2 mRNA levels (Fig 1C).

To validate this finding, we analyzed 4 matched diagnostic-remission bone marrow samples to determine the role of RBP2 in ALL progression. Each paired sample was obtained from the same patient at the time of initial diagnosis and complete remission. qRT-PCR of these samples revealed that RBP2 was significantly downregulated by the time that the patients underwent complete remission (Fig 1D, P<0.05). This suggested that RBP2 was overexpressed in both relapsed and *de novo* ALL samples, thereby revealing that RBP2 overexpression is correlated with the progression and leukemic burden of ALL.

### RBP2 is highly expressed in ALL cell lines

In addition to the clinical samples, we determined the expression patterns of RBP2 in ALL cells. Two T-lineage ALL (T-ALL) cell lines, Jurkat and SupT1, and one B-lineage ALL (B-ALL) cell line, Nalm6, were evaluated along with the bone marrow samples from two healthy patients that served as controls. Compared to the levels of RBP2 mRNA and protein observed in control patients, RBP2 mRNA and protein were highly expressed in ALL cells, especially in Jurkat cells (Fig 1E and 1F). Therefore, Jurkat cells were selected to further explore the potential oncogenic mechanism of RBP2 in ALL.

### RBP2 knockdown induces cell apoptosis and inhibits cell proliferation

**Fig 2:**
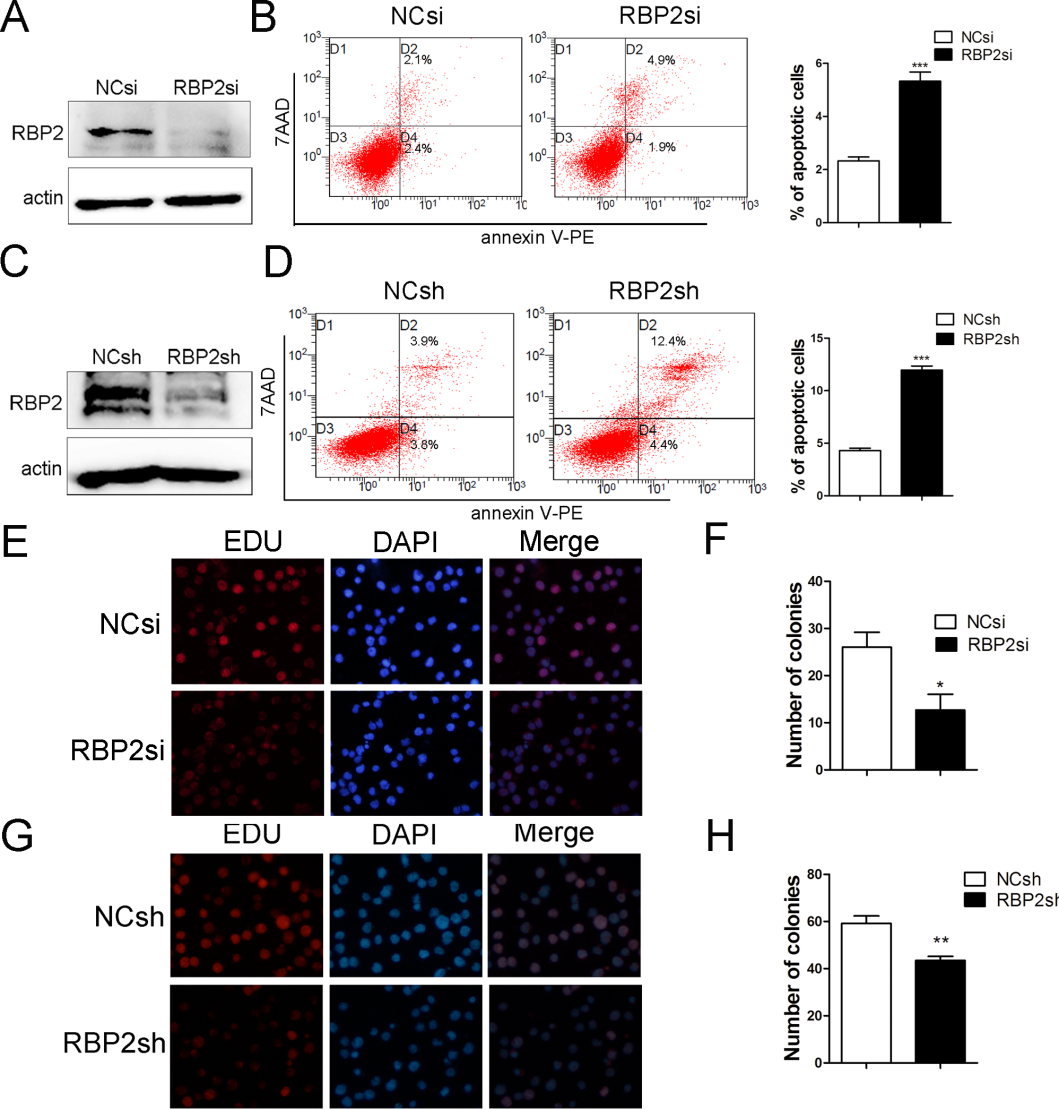
Effect of RBP2 knockdown on ALL apoptosis and cell viability. (A, C) RBP2 expression was measured by western blotting. (B, D) Flow cytometry analysis of the effect of RBP2 knockdown on apoptosis of Jurkat cells. The error bars represent SEM of 3 independent experiments. ***P< 0.001 by Student’s t-test. (E-H) Cell proliferation was measured by colony formation and EdU. The data are presented as the mean± SEM. *P< 0.05, **P< 0.01 by Student’s t-test.

To further analyze the function of RBP2, we transfected Jurkat cells with specific RBP2-siRNA and lenti-RBP2-shRNA virus. After a 72h-incubation, the proportion of apoptotic Jurkat cells was determined by flow cytometry. RBP2 expression was examined by western blotting (Fig 2A–2D). As expected, RBP2 knockdown resulted in a significant increase in apoptosis of Jurkat cells (Fig 2A–2D). We next sought to determine whether RBP2 knockdown has deleterious effects on cell viability. As expected, ALL cells treated with RBP2-specific siRNA and lenti-RBP2-shRNA consistently showed a decrease in cell proliferation and colony formation ability (Fig 2E–2H). Thus, RBP2 knockdown induced apoptosis and inhibited proliferation of ALL cell lines.

### BCL2 is a novel target gene of RBP2

**Fig 3:**
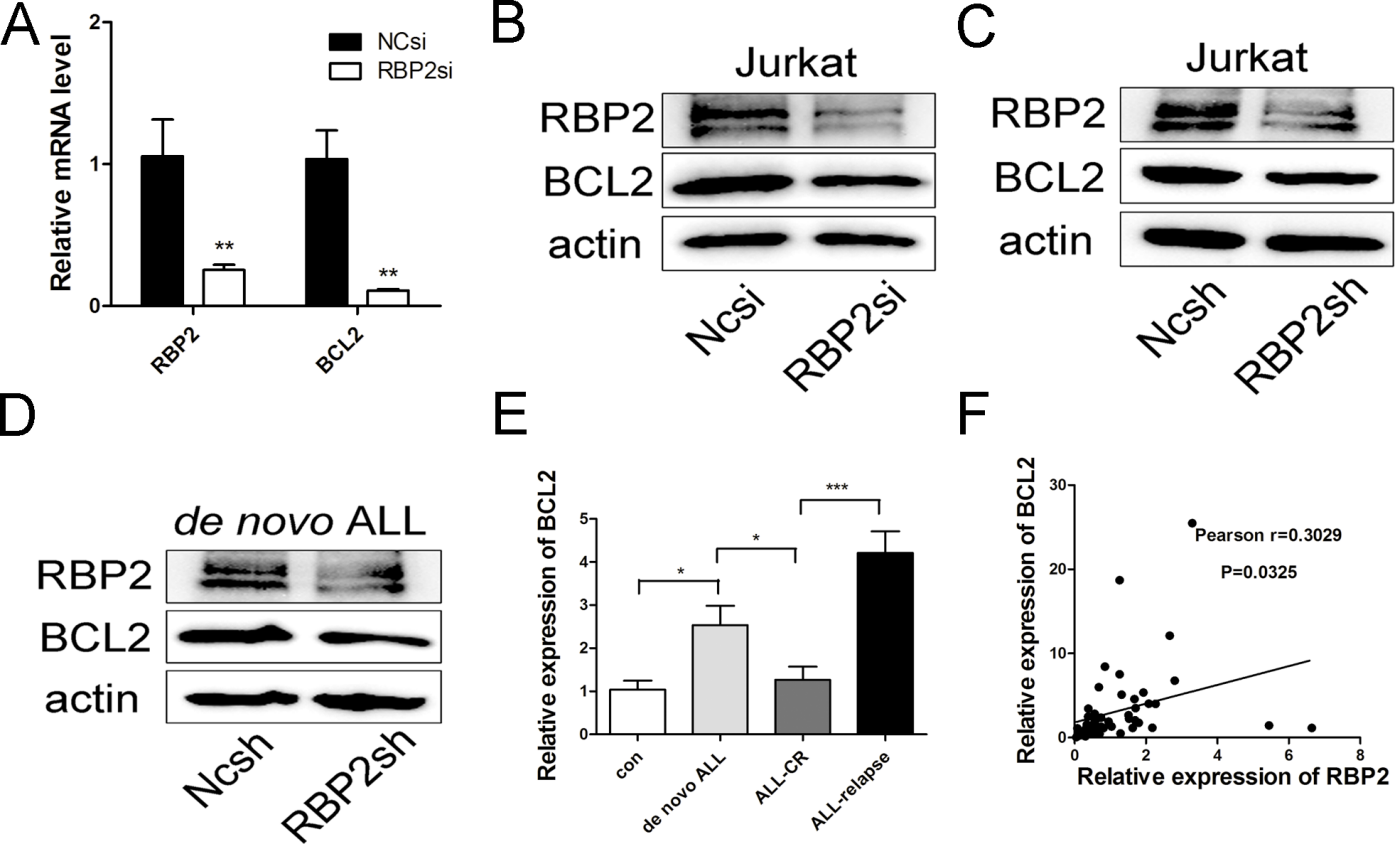
Effect of RBP2 on BCL2 expression. (A) qRT-PCR analysis of RBP2 and BCL2 mRNA levels after transfection with RBP2-siRNA in Jurkat cells for 72h. Data are presented as the mean± SEM. **P< 0.01 by Student’s t-test. (B, C) Western blotting analysis of RBP2 and BCL2 protein levels after transfection with RBP2-siRNA and RBP2-shRNA in Jurkat cells. (D) Western blotting analysis of RBP2 and BCL2 protein levels after transfection with lenti-RBP2-shRNA virus in ALL primary cells for 72h. The primary cells were from the bone marrow of ALL patients. (E) BCL2 mRNA levels in ALL samples. Mann-Whitney U-test was used to calculate the P value;*P< 0.05, ***P< 0.01. (F) The correlation between RBP2 and BCL2 in ALL samples (Pearson r = 0.3029, n = 50); *P< 0.05, ***P< 0.001.

Given that RBP2 was a regulator of apoptosis in Jurkat cells, we aimed to determine the targets of RBP2 that induced apoptosis. As a well-established inhibitor of mitochondrial apoptotic pathways, BCL2 has emerged as a potential therapeutic target in leukemias. After Jurkat cells were transfected with RBP2-siRNA and lenti-RBP2-shRNA, we found that BCL2 mRNA and protein levels were significantly reduced (Fig 3A–3C). We also separated bone marrow cells from *de novo* ALL patients and transfected primary ALL cells with specific lenti-RBP2-shRNA. The results showed that RBP2 depletion reduced BCL2 expression in ALL primary cells (Fig 3D).

To further explore the correlation between RBP2 and BCL2, we explored their mRNA expression levels in ALL samples (Figs 1C and 3E). As expected, their mRNA levels were positively correlated (Fig 3F). Thus, these results suggested that BCL2 might be a novel target gene of RBP2.

### RBP2 directly targets the promoter of BCL2

**Fig 4:**
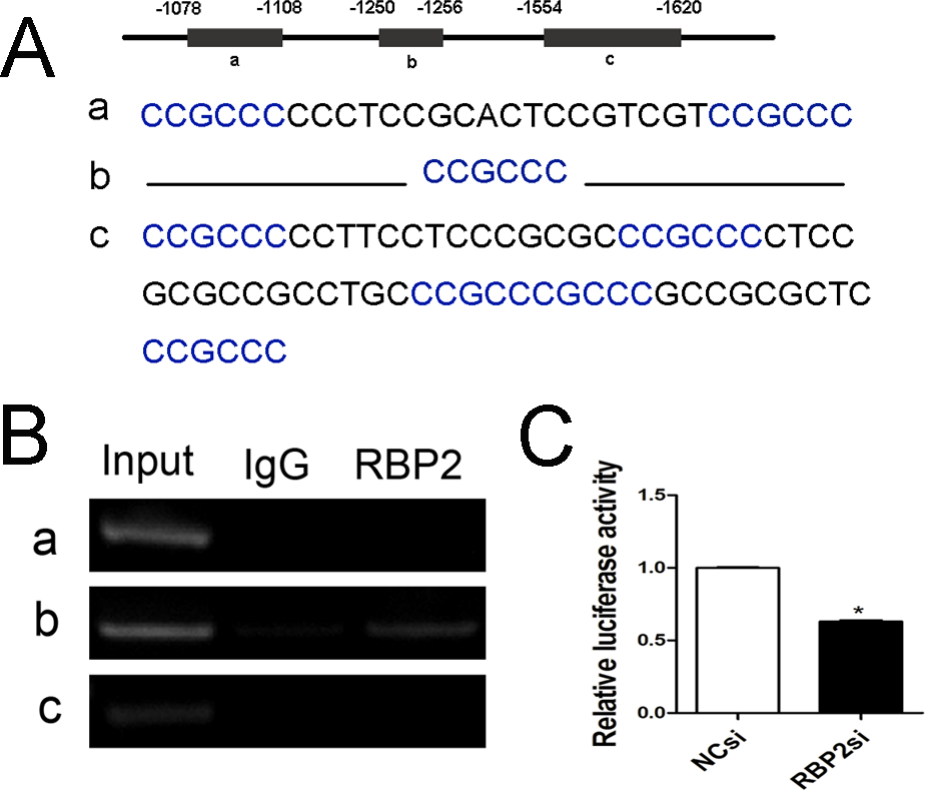
RBP2 directly targets the promoter of BCL2. (A) Predicted binding sites of RBP2 in the BCL2 promoter (-1078~-1620) were divided into regions a, b, and c. (B) ChIP assay for binding of RBP2 to BCL2 promoter regions a, b, and c. (C) Luciferase activities of the BCL2 promoter were determined after RBP2-siRNA transfection for 72 h and were normalized by Renilla luciferase activity. The data represent the mean ± SEM (standard error of the mean). An unpaired t-test was performed for statistical analyses; *P< 0.05.

To further explore whether BCL2 is a direct target of RBP2, we analyzed the promoter sequence of BCL2. Surprisingly, we found 8 binding motifs (CCGCCC) in the promoter region. We divided them into regions a, b, and c (Fig 4A). To determine whether RBP2 could bind to the human BCL2 promoter in intact cells, we performed a CHIP assay in Jurkat cells. We found that RBP2 bound to the b region but not to the a or c regions (Fig 4B). To determine the effect of RBP2 on BCL2 promoter activity, we transfected Jurkat cells with RBP2-siRNA on day 1 and wild-type BCL2 promoter reporter plasmid on the following day. Upon RBP2 knockdown, BCL2 promoter activity was significantly decreased (Fig 4C). Therefore, RBP2 bound to the promoter of BCL2 and affected its transcriptional activity.

### BCL2 mediates RBP2-induced proliferation and inhibition of apoptosis, and RBP2 inhibition sensitizes Jurkat cells to ABT-199

**Fig 5:**
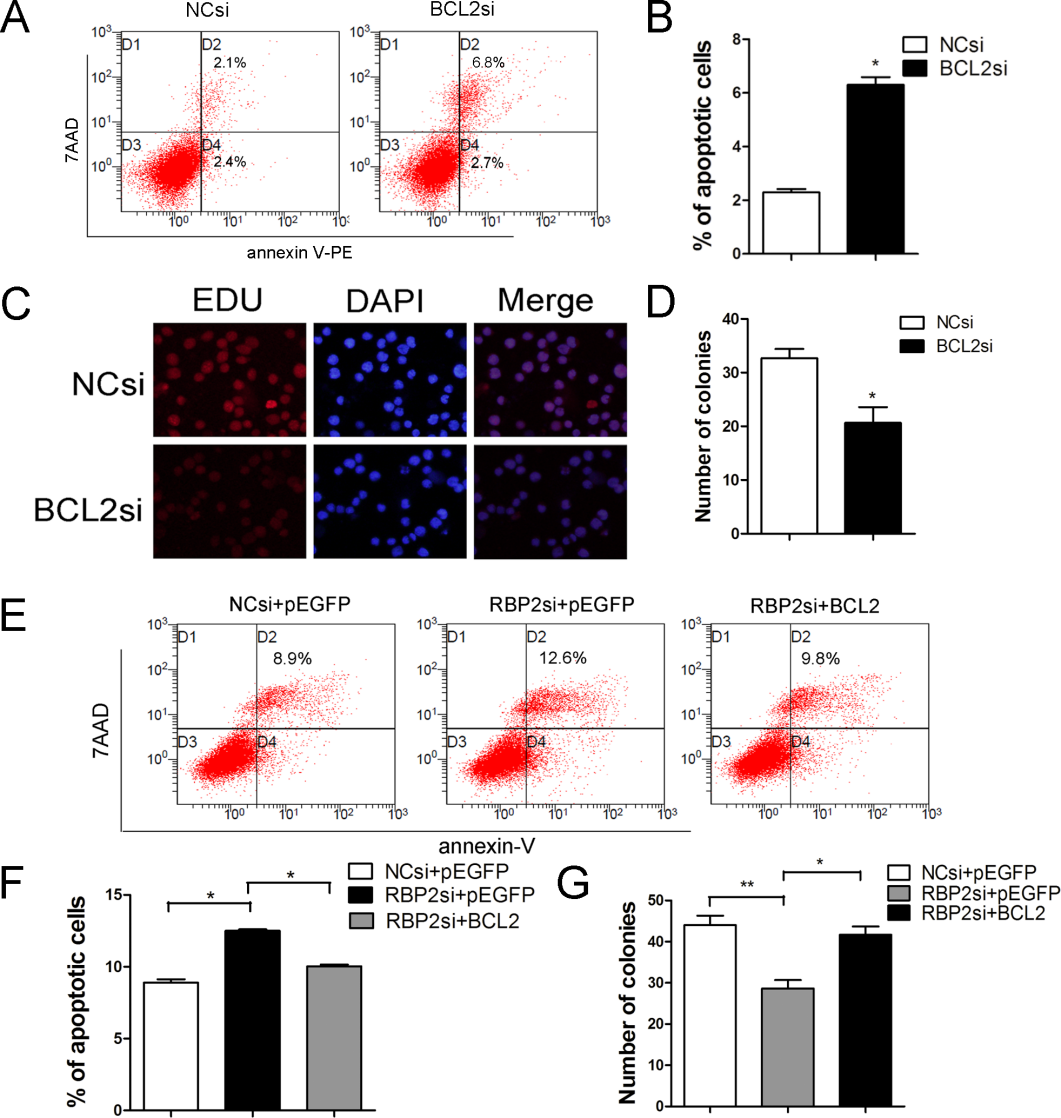
Effect of BCL2 on RBP2-mediated apoptosis and cell viability. (A, B) Flow cytometry analysis of BCL2 knockdown on the apoptosis of Jurkat cells. *P< 0.05 by Student’s t-test. (C, D) Cell proliferation after BCL2 knockdown was measured by EdU and colony formation. *P< 0.05 by Student’s t-test. (E, F) Flow cytometry analysis of apoptosis in Jurkat cells after RBP2-siRNA transfection with or without the BCL2 expression plasmid for 72 h. *P< 0.05 by Student’s t-test (G) Colony formation assay of Jurkat cells after RBP2-siRNA transfection with or without the BCL2 expression plasmid for 72 h. *P< 0.05, **P< 0.01 by Student’s t-test. The results are from 3 independent experiments.

**Fig 6:**
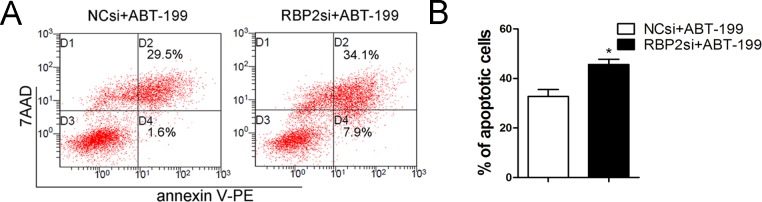
RBP2 knockdown sensitized Jurkat cells to ABT-199. (A, B) Flow cytometry analysis of apoptotic cells induced by 2 μM ABT-199 with or without RBP2-siRNA knockdown in Jurkat cells. The data represent the mean ± SEM (standard error of the mean). An unpaired t-test was performed for statistical analyses; *P< 0.05.

To study the functional contribution of BCL2 to the RBP2 knockdown-induced phenotype, we transfected BCL2 siRNA or control siRNA into Jurkat cells. We found that BCL2 depletion induced apoptosis and inhibited proliferation of Jurkat cells (Fig 5A–5D). Then, Jurkat cells were treated with both RBP2-siRNA and a BCL2 expression plasmid; a higher number of apoptotic cells were detected by flow cytometry than without transfection of the BCL2 expression plasmid (Fig 5E and 5F). Additionally, RBP2 knockdown-induced proliferation inhibition was also reversed after BCL2 expression (Fig 5G). These results showed that BCL2 overexpression significantly abrogated the induced apoptosis and inhibited proliferation mediated by RBP2 depletion (Fig 5E–5G). Therefore, induction of apoptosis and suppression of proliferation by RBP2 depletion partially depends on the repression of BCL2. Furthermore, RBP2 depletion increased the sensitivity of Jurkat cells to the BCL2 inhibitor ABT-199 (Fig 6A and 6B).

## Discussion

In this study, we identified a critical function of RBP2 in mediating apoptosis and proliferation in ALL. Our data indicated that RBP2 played an oncogenic role in adult ALL and that its overexpression correlated with the progression of ALL. Essentially, we found that the anti-apoptosis protein BCL2 was a novel target of RBP2 and mediated the role of RBP2. Our data supported the notion that RBP2 regulated the expression of BCL2 by directly binding its promoter. These findings suggested that RBP2 overexpression played a critical role in the development and progression of adult ALL by targeting BCL2.

RBP2 has been implicated as an oncoprotein in various solid cancers. In gastric cancer, its inhibition triggers cellular senescence [17] and promotes angiogenesis [18] and malignant transformation [19, 20]. RBP2 is also critical for breast cancer progression and metastasis [21]. In addition, RBP2 is involved in the carcinogenesis and progression of lung cancer [16]. However, we have shown that RBP2 is downregulated in the blast crisis of CML and that RBP2 plays an anti-oncogenic role [24]. Recently, JARID1B/KDM5B, another member of the JARID protein family, has been shown to be upregulated by Ikaros, HDAC1 and CK2 in B-cell ALL [25]. Such evidence suggests that the mechanisms of epigenetic dysregulation are complexly involved in malignancies. In this regard, exploring the novel functions and the mechanisms of RBP2 is imperative. In this study, we aimed to explore the role of RBP2 in ALL. Our results demonstrated that RBP2 overexpression was common in newly diagnosed ALL and relapsed patients. As expected, RBP2 expression decreased after treatment in patients who achieved complete remission. Thus, RBP2 overexpression is not only involved in the leukemogenesis of ALL but its overexpression might indicate ALL relapse. These results lay the foundation and present further questions for future research. Here, we found that RBP2 depletion induced apoptosis and inhibited the cell viability of ALL cells. Thus, as a novel finding for this field of scientific research, we established the role of RBP2 in apoptosis.

BCL2, an inhibitor of the mitochondrial apoptosis pathway, is the founding member of a large family of BH-domain-containing proteins with activities upstream of caspase activation.BCL2 has emerged as a potential therapeutic target in both leukemia [26, 27] and solid tumors [28]. As a breakthrough finding, our experiments are the first to demonstrate that BCL2 is a novel target of RBP2. The AT-rich interaction domain of RBP2 can recognize the specific DNA sequence CCGCCC contained in the promoter regions [29]. We identified 8 binding motifs in the BCL2 promoter and divided them into three regions (a, b, and c). Then, we found that RBP2 bound to the b region but not the a or c regions. RBP2 depletion significantly downregulated BCL2 expression and inhibited its promoter activity. Clearly, BCL2 is a new target gene of RBP2, which might mediate a new function of RBP2.

H3K4 methylation is usually associated with transcriptional activation. RBP2 specifically targets tri- and di-methylated lysine 4 of histone H3 (H3-K4) for demethylation and acts as a transcriptional repressor at its target promoters. However, we found that RBP2 directly bound to the BCL2 promoter and induced its transcriptional activation. *DiTacchio L* found that RBP2 increased histone acetylation by inhibiting histone deacetylase 1 function and enhanced transcription via CLOCK-BMAL1 in a demethylase-independent manner [30]. In gastric cancer, *Li L* also found that RBP2 activatesVEGF [18]. These results support models in which RBP2 mediates the transition from repression to robust activation of target genes. Therefore, more evidence and additional studies will be required to determine the acetylation status of the BCL2 promoter and the interaction of RBP2 with histone deacetylase 1 and transcriptional factors such as CLOCK-BMAL1.

The inhibitors of BCL2 show efficacy in pre-clinical and early phase clinical trials of myeloma [31], small-cell-lung cancer [32], high-risk B cell non-Hodgkin lymphoma, and leukemia [26–27, 33–34]. As targeting the anti-apoptotic pathway provides opportunities for target therapy, we explored the synthetic role of the BCL2 inhibitor ABT-199 and RBP2 inhibition. Our results showed that RBP2 depletion sensitized Jurkat cells to ABT-199. These data indicate that the combination of ABT-199 and RBP2 depletion may allow ABT-199 to be used at a low concentration.

Our studies have demonstrated the oncogenic role of RBP2 in adult ALL. In addition, we have verified that RBP2 affects apoptosis by targeting BCL2. Remarkably, RBP2 plays contrasting roles in ALL and myeloid leukemia. Thus, RBP2 might serve as a potential diagnostic marker for different types of leukemia. These results emphasize the need for future investigation on the rather opposite roles that RBP2 plays in lymphoid and myeloid leukemia.

## Supporting Information

S1 Fig Uncropped gels for Figs 1 and 2. (TIF)

S2 Fig Uncropped gels for Figs 3 and 4. (TIF)

S1 Table The clinical characteristics of all ALL patients. (DOCX)

S1 File Underlying participant-level data in the article. (XLSX)
