# Supplementary figures and images for: RBP2 Promotes Adult Acute Lymphoblastic Leukemia by Upregulating BCL2
Source: PLoS One. 2016 Mar 23;11(3):e0152142. doi: 10.1371/journal.pone.0152142 (PMC4805198; doi:10.1371/journal.pone.0152142)

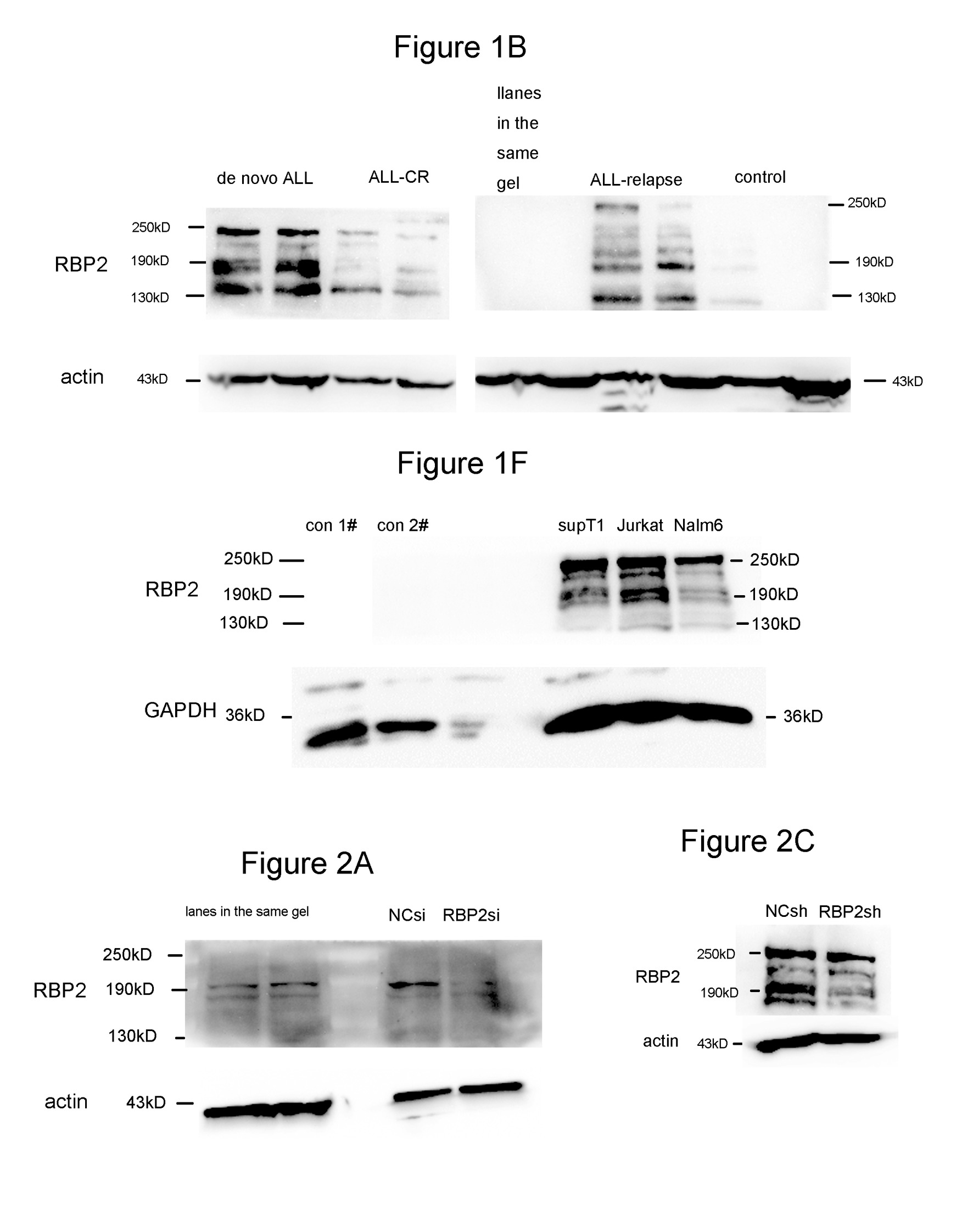

Supplement: S1 Fig — (TIF) [file pone.0152142.s001.tif]

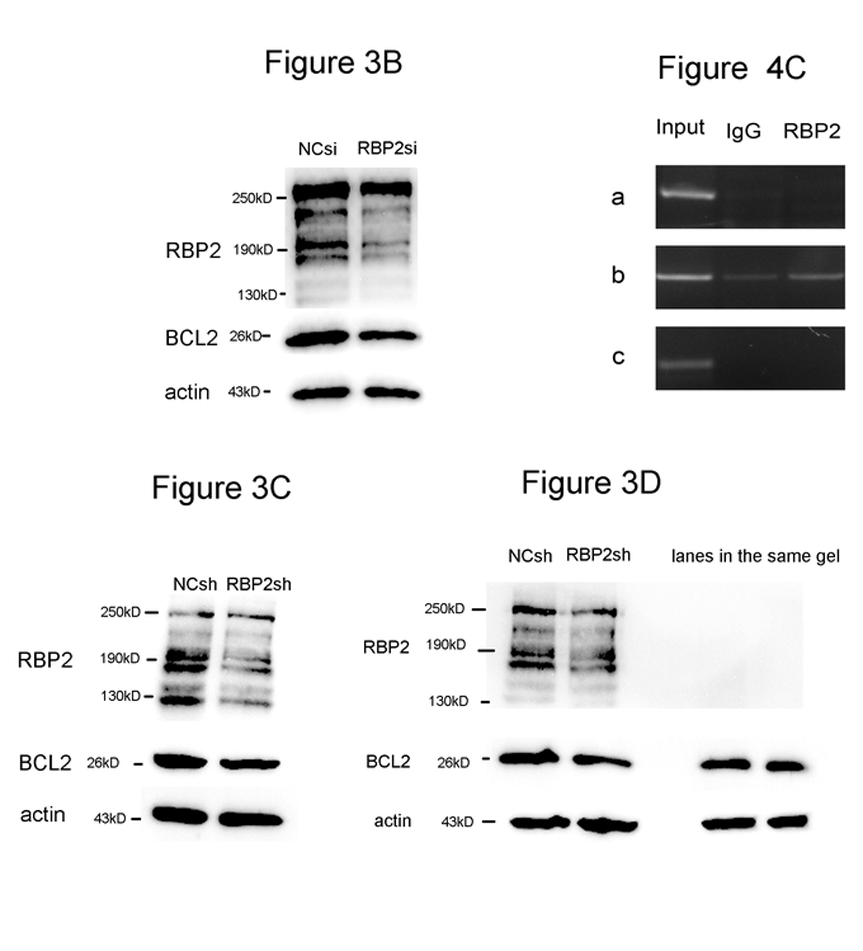

Supplement: S2 Fig — (TIF) [file pone.0152142.s002.tif]
