# Supplementary material for: RBP2 Promotes Adult Acute Lymphoblastic Leukemia by Upregulating BCL2
Source: PLoS One. 2016 Mar 23;11(3):e0152142. doi: 10.1371/journal.pone.0152142 (PMC4805198; doi:10.1371/journal.pone.0152142)
Supplement: S1 Table — (DOCX) [file pone.0152142.s003.docx]

| **Characteristic** | | **ALL**  **(n=42)** | **ALL-CR**  **(n=36)** | **ALL-relapse**  **(n=9)** |
| --- | --- | --- | --- | --- |
| **Gender** | Male | 26 | 22 | 4 |
|  | Female | 16 | 14 | 5 |
| **Age(years)** | Median | 34 | 35 | 37 |
|  | Range | 18-70 | 18-65 | 18-57 |
| **WBC,×109/L** | Median | 71.63 | 3.43 | 43.83 |
|  | Range | 1.32-399.19 | 0.13-6.45 | 1.51-204.61 |
| **Hemoglobin, g/L** | Median | 90.1 | 97.9 | 100.7 |
|  | Range | 35-163 | 67-116 | 81-150 |
| **Platelet count, ×109/L** | Median | 73.0 | 292.2 | 130.17 |
|  | Range | 1-351 | 10-876 | 25-333 |
| **Immunophenotype** | B-ALL | 34 | 29 | 7 |
|  | T-ALL | 8 | 7 | 2 |
| **BCR-ABL** | + | 9 | 9 | 2 |
|  | - | 33 | 27 | 7 |

**S1 Table. The clinical characteristics of all ALL patients**
